# Supplementary material for: Oscillatory Coupling Between Thalamus, Cerebellum, and Motor Cortex in Essential Tremor
Source: Mov Disord. 2025 Mar 3;40(5):896–905. doi: 10.1002/mds.30165 (PMC12089897; doi:10.1002/mds.30165)
Supplement: Supplementary file 1 — Data S1. Supporting Information. [file MDS-40-896-s001.docx]

**Oscillatory coupling between thalamus,**

**cerebellum and motor cortex in essential tremor**

**Supplementary material**

Alexandra Steina^1^, Sarah Sure^1^, Markus Butz^1^,
Jan Vesper^2^, Alfons Schnitzler^1^, Jan Hirschmann^1^

**Author affiliations:**

1 Institute of Clinical Neuroscience and Medical Psychology, Medical Faculty, Heinrich Heine University, 40225, Düsseldorf, Germany

2 Department of Functional Neurosurgery and Stereotaxy, Neurosurgical Clinic, Medical Faculty, Heinrich Heine University, 40225, Düsseldorf, Germany

**Supplementary Table 1 Task & tremor information.**

| **Patient ID** | **Tasks performed** | **Postural tremor frequency L/R [Hz]** | **Kinetic tremor frequency L/R [Hz]** | **Postural tremor data length L/R [s]** | **Kinetic tremor data length L/R [s]** |
| --- | --- | --- | --- | --- | --- |
| ET01 | H, P | - | - | - | - |
| ET02 | H | 4.5 / 4.5 | - | 88 / 78 | - |
| ET03 | H | 5 / 5.5 | - | 99 / 40 | - |
| ET04 | H | - | - | - | - |
| ET05 | H | 6 / - | - | 153 / - | - |
| ET06 | H | 6.5 / 6.5 | - | 81 / 80 | - |
| ET07 | H | - | - | - | - |
| ET08 | H | 5 / 5.5 | - | 297 / 213 | - |
| ET09 | P | - | 5 / 5.5 | - | 81 / 72 |
| ET10 | H | - | - | - | - |
| ET11 | H, P | - | 3 / 5 | - | 63 / 109 |
| ET12^a^ | H, P | 5.5 / - | 6.5 / - | 236 / - | 102 / - |
| ET13 | H, P | - | - | - | - |
| ET14 | H, P | - | 4.5 / 3.5 | - | 130 / 146 |
| ET15 | H, P | 5 / - | 5 / 4.5 | 174 / - | 128 / 109 |
| ET16 | H, P | - | - | - | - |
| ET17 | H | 3.5 / 3.5 | 3.5 / - | 102 / 90 | 49 / - |
| ET18 | H | 5 / 4 | - | 69 / 65 | - |
| ET19 | H | 5.5 / 5.5 | - | 87 / 137 | - |
| $\mu\pm\sigma$ |  | 5.1$\pm$0.9 | 4.4 $\pm$ 1.1 | 122 $\pm70$ | 98.9 $\pm$ 32 |

H: Hold, B: Button press, P: Pour $\mu$: mean, $\sigma$: standard deviation.

^a^Left VIM excluded due to uncertain electrode position.

**Methods**

**Referencing scheme**


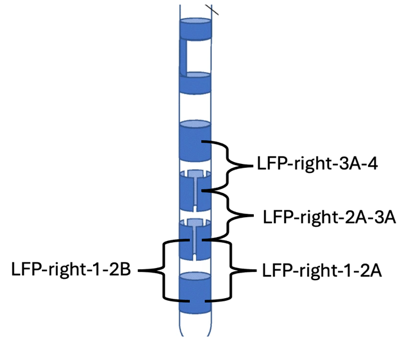


**Supplementary Fig. 1**: Bipolar montage used for re-referencing the contacts of a segmented lead. Each segment on level 2 was referenced to the corresponding segment on level 3, resulting in the bipolar channels 2A-3A, 2B-3C and 2C-3C. All segments were further referenced to the ring contact above (level 3) or below (level 2).

**Lead localization**

Lead localization was done in Lead-DBS v2.3, using pre-operative MRI and a post-operative CT scan. A linear co-registration of CT and MRI scans was performed with advanced normalization tools (ANTs).^1^ The coregistration was visually inspected and adjusted if necessary. Subsequently, the pre-operative images were normalized from subject space to MNI space (ICBM 152 2009b Nonlinear Asymmetric),^2^ with the ANTs-based diffeomorphic normalization SyN algorithms.^1^ A brain shift correction was performed, using a coarse mask by Schönecker.^3^ Lead trajectories were either automatically reconstructed with PACER^4^ and manually refined or fully manually reconstructed if PACER failed.

**Power spectra – FOOOF algorithm**

We applied the fitting oscillations and one over f (FOOOF) algorithm to model the periodic and aperiodic component of the LFP and EMG spectra. The model was fitted to the spectrum in the 2-35 Hz range. The aperiodic component was fitted with either a fixed or a knee model. The peak width parameter was set to 1-3 Hz and the number of Gaussian fits was ranging from 8-12. All spectra were visually inspected and corrected if necessary to ensure good model fits.

**Results**


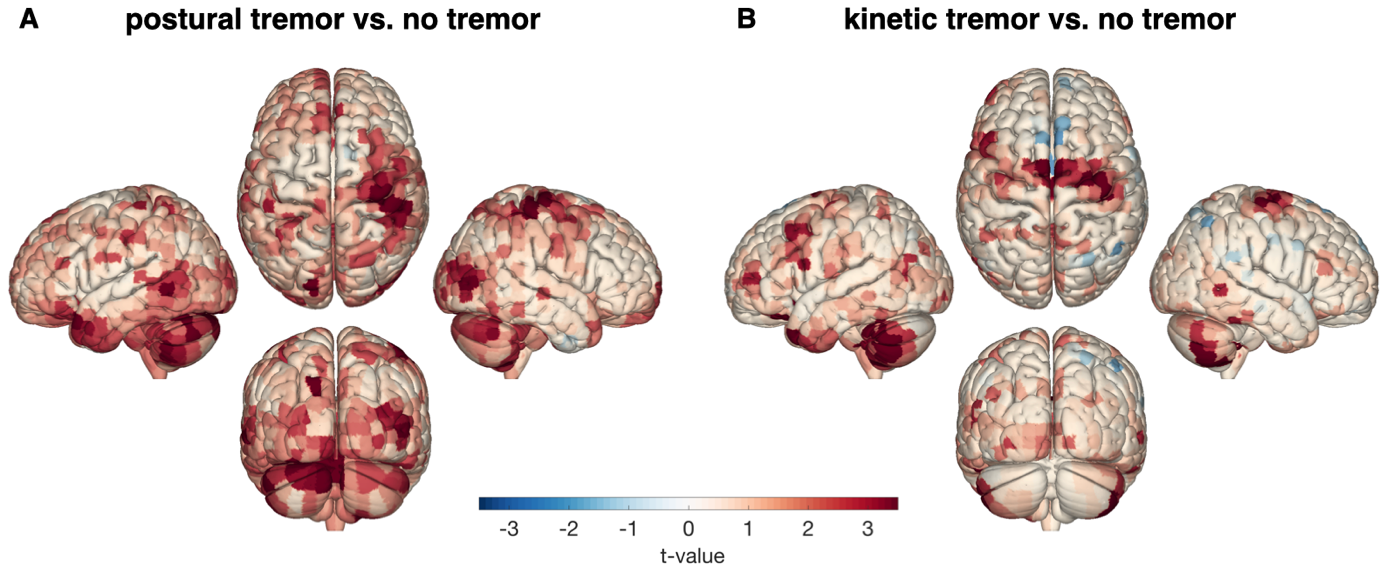


**Supplementary Fig. 2: *t*-maps of tremor vs. no tremor for thalamocortical coherence. A** Postural tremor vs. no tremor**. B** Kinetic tremor vs. no tremor. Cluster-based permutation test.


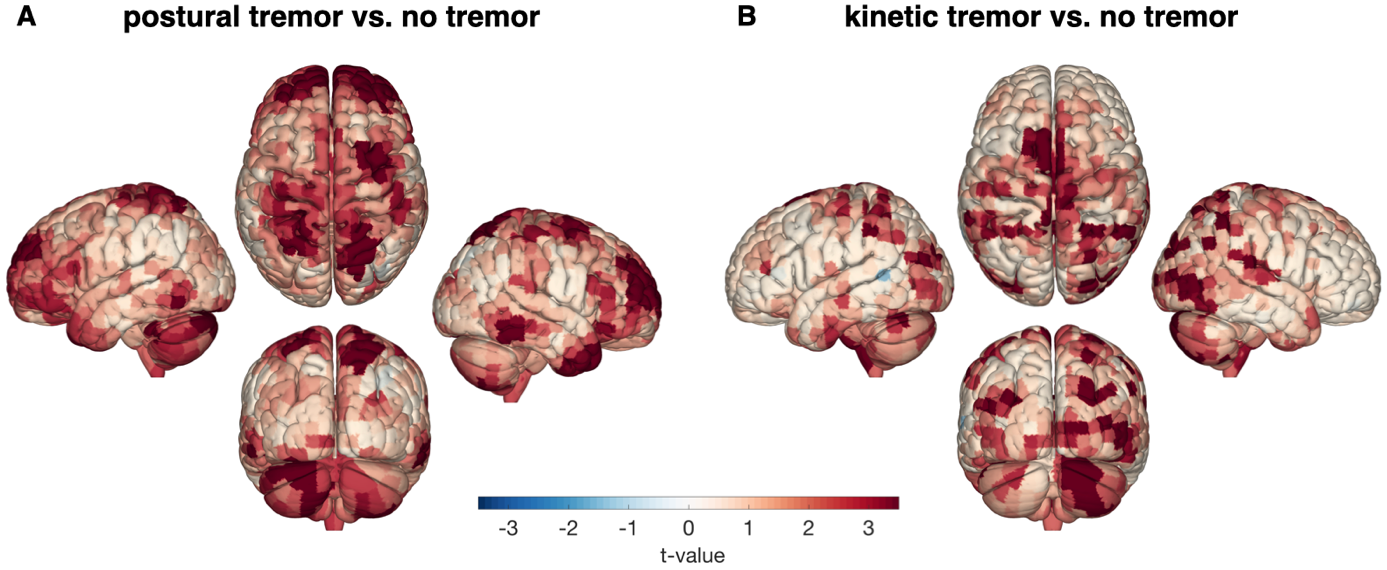


**Supplementary Fig. 3: *t*-maps of tremor vs. no tremor corticomuscular coherence. A** Postural tremor vs. no tremor. **B** Kinetic tremor vs. no tremor. Cluster-based permutation test.

**References**

1. Avants BB, Tustison NJ, Song G, Cook PA, Klein A, Gee JC. A reproducible evaluation of ANTs similarity metric performance in brain image registration. *NeuroImage*. 2011;54(3):2033-2044. doi:10.1016/j.neuroimage.2010.09.025

2. Fonov V, Evans AC, Botteron K, Almli CR, McKinstry RC, Collins DL. Unbiased average age-appropriate atlases for pediatric studies. *NeuroImage*. 2011;54(1):313-327. doi:10.1016/j.neuroimage.2010.07.033

3. Schönecker T, Kupsch A, Kühn AA, Schneider GH, Hoffmann KT. Automated Optimization of Subcortical Cerebral MR Imaging−Atlas Coregistration for Improved Postoperative Electrode Localization in Deep Brain Stimulation. *Am J Neuroradiol*. 2009;30(10):1914-1921. doi:10.3174/ajnr.A1741

4. Husch A, V. Petersen M, Gemmar P, Goncalves J, Hertel F. PaCER - A fully automated method for electrode trajectory and contact reconstruction in deep brain stimulation. *NeuroImage Clin*. 2018;17:80-89. doi:10.1016/j.nicl.2017.10.004
